# Self-worth, emotional labor, work engagement, and anxiety among nurses: a latent profile and mediation analysis

**DOI:** 10.3389/fpubh.2026.1782042

**Published:** 2026-04-09

**Authors:** Jiayi Gu, Rui Zeng, Lijuan Quan, Hongjuan Chang

**Affiliations:** 1 Faculty of Medicine, Wuhan University of Science and Technology, Wuhan, China; 2 Department of Nursing, The First Affiliated Hospital, Zhejiang University School of Medicine, Hangzhou, China

**Keywords:** anxiety, emotional regulation, nurses, self concept, work engagement

## Abstract

**Background:**

Nurse anxiety threatens care quality and workforce stability. The objective of this study was to examine the relationship between self-worth and anxiety among nurses, and to investigate the chain mediating roles of work engagement and emotional labor across heterogeneous subgroups.

**Methods:**

Using a cross-sectional design and random sampling, a questionnaire survey was conducted among 1,280 nurses from a Grade A tertiary hospital in Wuhan from March to August 2025. The measurement tools included a General Information Questionnaire, the Adult Self-Worth Questionnaire, the Generalized Anxiety Disorder Scale, the Work Engagement Scale, and the Emotional Labor Scale. Latent Profile Analysis (LPA) was applied to identify heterogeneous subgroups. PLS-SEM was used to examine the relationships between nurses' self-worth and anxiety (with work engagement and emotional labor as mediating variables).

**Results:**

Three nurse subgroups were identified: the “Resource-Depleted Group” (19.38%), the “Balanced Group” (69.92%), and the “Resource-Abundant Group” (10.70%). The anxiety scores of the three subgroups were (7.51 ± 4.61), (7.16 ± 5.16), and (6.46 ± 4.43) (mean ± SD), respectively, showing statistically significant differences (*F* = 7.85, *p* < 0.050). Hierarchical mediation analysis revealed that the chain mediation effect was non-significant in the Resource-Depleted Group, where only professional self-worth exerted a direct negative predictive effect on anxiety (β = −1.1047, *p* < 0.050). In contrast, significant chain mediation effects were observed in both the Balanced Group and Resource-Abundant Group (both *p* < 0.050).

**Conclusion:**

**:** Three subgroups with significantly distinct resource states were identified among nurses: the Resource-Depleted Group, the Balanced Group, and the Resource-Abundant Group. Moreover, the effect of self-worth on nurses' anxiety through work engagement and emotional labor was found to be subgroup-dependent.

## 1 Introduction

Anxiety is a complex psychophysiological state encompassing cognitive, somatic, emotional, and behavioral dimensions. It is characterized by subjective experiences of distress, unease, worry, fear, or apprehension, often accompanied by physiological and behavioral manifestations such as sleep disturbances and autonomic hyperarousal (1).

As a normal psychological response to stress, moderate anxiety can enhance individual adaptability by motivating coping behaviors in challenging situations. However, failure to manage anxiety effectively may lead to pathological conditions involving headaches, diaphoresis, muscle tension, palpitations, fatigue, and even exhaustion. Pathological anxiety, as a persistent and excessive emotional disorder, significantly impairs individual functioning, behavior, interpersonal relationships, and overall quality of life (2). Anxiety symptoms are prevalent across various occupational settings, with healthcare environments being particularly high-risk due to their inherently stressful and demanding nature (3). Nurses constitute the largest workforce in healthcare systems worldwide and play an indispensable role in ensuring patient safety and delivering high-quality care (4). Nevertheless, the nature of nursing work exposes this population to chronic high-intensity occupational stressors, including circadian rhythm disruption from rotating shift work (5), excessive workload exceeding physical and psychological capacity (6), psychological depletion from role conflict (7), and psychological trauma resulting from workplace violence (8). The cumulative effect of these occupational hazards significantly increases nurses' vulnerability to adverse mental health outcomes, including anxiety, depression, and fatigue (9–11). Such psychological distress may subsequently precipitate job burnout, increase the risk of medical errors, and ultimately compromise the quality of healthcare services (12). A survey involving 4,188 Chinese nurses during the COVID-19 pandemic revealed that the prevalence of anxiety was as high as 46% (13), positioning nurses' mental health as a critical concern in healthcare management. In high-stress units such as emergency departments and intensive care units, the detection rate of anxiety disorders among nurses is three to five times higher than that of the general population, serving as a primary driver of occupational attrition (14). This psychological distress, through its cascading effects such as job burnout and workforce turnover, further exacerbates the global shortage of nursing personnel (15). For healthcare systems already constrained by workforce deficits, addressing the occupational health and psychological wellbeing of nurses is not only a manifestation of humanitarian concern but also an inherent requirement for building a sustainable and high-quality healthcare system. Therefore, investigating protective factors against anxiety and identifying precise and effective intervention pathways have emerged as critical research priorities in the nursing field.

Notably, the occurrence and development of anxiety among nurses are influenced not only by external work environments but also by individual internal psychological factors. Among these, self-worth, as a core psychological trait, is closely associated with nurses' anxiety (16, 17). A systematic review encompassing multiple countries indicates that self-worth, as a key protective factor for mental health, can effectively buffer the negative impact of occupational stress on nurses' emotions (18, 19). However, its predictive role in anxiety has not been conclusively validated, and research on the underlying mechanisms remains insufficient.

Work engagement is a positive work-related state characterized by vigor, dedication, and absorption. It manifests as high energy levels, strong adaptability, a profound sense of meaning and motivation derived from work, and complete immersion in one's tasks (20). Previous studies have demonstrated that increased work engagement among nurses is associated with reduced patient mortality and complication rates (21). Therefore, investigating the mechanisms of work engagement in nurses is of great significance. However, existing research has not reached a consensus on the level of work engagement among nurses. For instance, a study conducted in Spain reported high levels of work engagement among nurses (22), whereas another study focusing on Chinese nurses found moderate levels of work engagement (23). Nevertheless, these studies did not establish consistency in nurses' work engagement. In their daily practice, nurses must provide high-quality services through continuous nurse-patient interaction (24). The literature indicates that most nurses employ emotional labor, regulating their emotional expressions to align with patient experiences (25). Emotional labor refers to the process of managing feelings and expressions to fulfill organizational expectations (26). With the growing demand for high-quality medical services, healthcare institutions have increasingly emphasized the provision of patient-centered care (24). In South Korea, for example, many hospitals regulate nurses' behaviors and facial expressions to comply with organizational standards and ensure service quality (27). Consequently, emotional labor among nurses is regarded as an essential component of patient care (28).

Based on this background, this study explores the relationships among self-worth, work engagement, emotional labor, and anxiety in Chinese nurses. We proposed and tested research hypotheses using survey data. This is the first study to employ a chained mediation model to examine these variables in Chinese nurses. Additionally, latent profile analysis (LPA) was used to classify nurses and explore differences in anxiety across subgroups.

## 2 Literature review and research hypotheses

### 2.1 Self-worth

Self-worth refers to an individual's self-cognition and evaluation, encompassing both positive and negative self-perceptions. It represents one's attitude toward and judgment of oneself, essentially functioning as a positive self-affective experience (29). Self-worth exerts extensive influence on individual emotions, cognition, volition, and behavior, and is inextricably linked to mental health status (30). Its level directly affects psychological adaptability when facing stress, thereby regulating the generation and development of negative emotions (31). Sociometer theory posits that self-worth serves as a sociometer, motivating individuals to alleviate negative emotions such as anxiety, worry, and depression through cognitive adjustment or behavioral action (32). Specifically, nurses with high self-worth tend to maintain positive perceptions of their professional competence, contributions to patients, and the meaning of their occupation. This positive self-perception helps them sustain psychological stability and adaptability when confronting occupational stress. Conversely, nurses with low self-worth may be more prone to self-doubt and self-negation, thereby falling into anxiety under stressful conditions. A cross-sectional study among Chinese nurses indicated that self-worth directly influences nurses' perceptions of their professional competence and is closely associated with psychological adaptability. Nurses with higher self-worth scores demonstrated stronger psychological adaptability and lower anxiety prevalence, with self-worth indirectly influencing the development of anxiety through its regulation of psychological adaptability (33). A meta-analysis synthesizing studies (34) from seven countries revealed that nurses' occupational benefit perception is positively correlated with self-worth. The professional recognition and self-actualization experiences derived from perceived occupational benefits can effectively enhance self-worth, thereby indirectly alleviating anxiety. Therefore, this study proposes Hypothesis 1 (H1): nurses' self-worth negatively predicts their anxiety.

### 2.2 Work engagement

The concept of work engagement was first introduced by American scholar Kahn (35) in 1990, defined as “the harnessing of organizational members' selves to their work roles through self-regulation to achieve a perfect integration of the self with a work role.” Work engagement represents a positive, fulfilling psychological state manifested by employees, reflecting their identification with, enthusiasm for, and immersion in work. Specifically, among nurses, those with higher work engagement enthusiastically devote more time and energy to their duties, achieve harmonious integration between self and work roles, and exhibit positive emotional, cognitive, and physical manifestations. This positive work state further reinforces their self-worth identification, reciprocally enhancing self-worth and consequently better buffering anxiety. Research has demonstrated that nurses' self-worth has a direct positive predictive effect on work engagement (30). Multiple studies have indicated a correlation between work engagement and anxiety. Peterson et al. (36) found that work engagement was positively associated with mental health outcomes, with healthcare workers reporting higher engagement exhibiting lower levels of anxiety and depression. However, Hallberg et al. (37) suggested that work engagement was negatively correlated with mental health problems. Nevertheless, the underlying mechanisms among self-worth, work engagement, and anxiety remain unclear. Based on previous empirical evidence, this study proposes Hypothesis 2 (H2): self-worth may enhance nurses' work engagement, thereby indirectly influencing anxiety.

### 2.3 Emotional labor

Scholars Hochschild (38) first proposed the concept of “emotional labor” in 1979, defining it as “the management of feelings to create a publicly observable facial and bodily display” required by organizational rules. According to Grandey's perspective (26), employees typically employ two emotional expression strategies: surface acting and deep acting—to meet organizational emotional display requirements. Surface acting involves superficial deception, where employees merely adjust their observable external emotional expressions to comply with organizational demands, without significantly altering their internal emotional experience; it constitutes a coping mechanism (39). Deep acting entails integrating one's genuine feelings, values, and inner experiences into the work. It is not merely a performance to meet organizational requirements but a natural expression of the individual's internal state. Since its introduction by Hochschild, the concept of emotional labor has been widely applied across various disciplines, including nursing. Soon after, it was introduced into the nursing field through studies examining how nursing students and ward nurses engage in emotion management to enhance patient care (40). Statistics indicate that 52.97% of nurses have experienced empathic emotions such as compassion and sadness in their work (41). For nurses, whose work primarily involves patients and their families, this professional characteristic determines the pervasiveness of emotional labor. Research has demonstrated that emotional labor exerts dual effects on both nurses and nursing organizations, particularly manifesting in nurses' physical and mental health outcomes (42). However, findings regarding these effects have been inconsistent across studies. On one hand, some research has identified negative consequences of emotional labor. For instance, Chen et al. (43) found that nurses often need to suppress their authentic emotions to provide professional services. Prolonged suppression may lead to heightened anxiety resulting from emotional labor, indicating a positive correlation between emotional labor and anxiety, though deep acting showed no significant association with anxiety. Conversely, other studies have reached different conclusions. Yilmaz et al. (44) reported that nurses' depression levels decreased as emotional labor increased, a negative association potentially attributable to the positive effects of deep acting. Grandey (45) also noted that deep acting generates more positive emotional experiences, better work performance, and greater job satisfaction. However, the relationship between emotional labor and mental health is not consistently uniform. Additionally, a Korean study (46) found that emotional labor was positively correlated with depression and negatively correlated with self-worth. These seemingly contradictory findings suggest that emotional expression requirements, surface acting, and deep acting are not mutually exclusive; individuals may simultaneously employ multiple forms of emotional labor to navigate complex situations (47). Therefore, examining the combined effects of these strategies may better reflect individuals' authentic emotional labor experiences and their health implications. Based on the aforementioned evidence, this study proposes Hypothesis 3 (H3): self-worth may indirectly influence anxiety by reducing nurses' emotional labor.

### 2.4 The relationship between work engagement, emotional labor, self-worth, and anxiety

Researchers have suggested that work engagement and emotional labor are also interrelated. Mauno et al. (48) proposed that higher emotional labor is associated with lower work engagement.

Multiple studies (49, 50) have found that nurses' work engagement correlates with emotional labor, with both surface acting and deep acting demonstrating negative correlations with work engagement. However, Zhou's research (50) notably found a positive correlation between work engagement and emotional labor. According to the Conservation of Resources (COR) Theory (51), individuals strive to acquire, retain, foster, and protect their personal resources. When resources are lost or threatened, they must be replenished promptly; otherwise, individuals will take measures to prevent resource depletion (52). Prolonged emotional labor and emotional burden not only affect nurses' work performance and job satisfaction but also impact their mental health (53). Firstly, limited research has focused on the relationships among self-worth, work engagement, emotional labor, and anxiety specifically in Chinese nurses. Secondly, the potential mediating mechanisms explaining these associations have been partially overlooked. Based on H2 and 3, whether work engagement and emotional labor sequentially mediate the relationship between self-worth and anxiety remains unclear. Therefore, this study proposes Hypothesis 4 (H4): work engagement and emotional labor may exert a chain mediating effect in the relationship between self-worth and anxiety.

### 2.5 Latent profile analysis (LPA)

To further elucidate the mechanism underlying the relationship between self-worth and nurses' anxiety, and to explore targeted intervention pathways for alleviating nurses' anxiety, this study incorporated work engagement and emotional labor as mediating variables and employed latent profile analysis (LPA) as an investigative approach. LPA is an emerging person-centered statistical method that explains associations among external continuous variables based on latent categorical variables, thereby achieving local independence among exogenous variables (54). Researchers worldwide have begun employing this method in relevant studies. For instance, one study (14) conducted latent profile analysis among emergency department nurses and identified significant internal differentiation in their mental workload, a finding that challenges the effectiveness of “one-size-fits-all” intervention strategies and suggests the need to analyze psychological risk generation mechanisms from a subgroup-specific perspective. Ma et al. (55) utilized LPA to categorize oncology nurses' work engagement into three types: highly efficient and focused, moderately balanced, and low-engagement coping. Additionally, Chen (52) and Zhou et al. (56), respectively applied this method to explore different types of work engagement and emotional labor among nurses. These studies provide an empirical foundation for the present study's in-depth analysis starting from the combination patterns of individual self-worth, work engagement, and emotional labor. In view of this, the present study proposes Hypothesis 5 (H5): self-worth, work engagement, and emotional labor form distinct configuration types across individuals. Within these different typological groups, the chained mediating effect of work engagement and emotional labor on the relationship between self-worth and anxiety exhibits significant heterogeneity.

**Figure 1:**
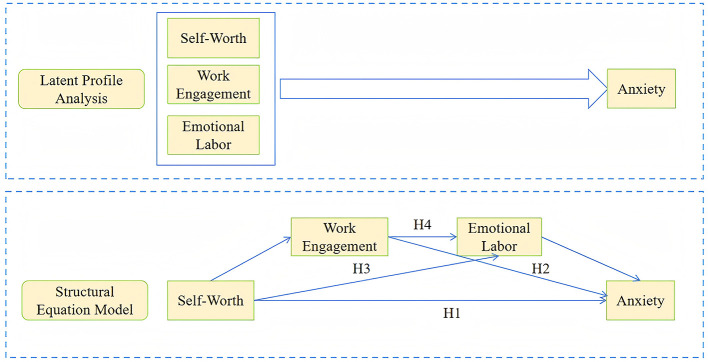
Study framework model.

Previous research on nurse anxiety has predominantly adopted variable-centered approaches; however, these methods focused on sample averages while neglecting within-group heterogeneity. Therefore, an integrated approach combining person-centered and variable-centered methodologies was employed in this study. Specifically, LPA was first conducted to explore how individual factors (self-worth, work engagement, and emotional labor) combined at the individual level. Subsequently, the predictive role of self-worth among different nurse types and the chain mediating effects of work engagement and emotional labor on the relationship between self-worth and anxiety across these types were further investigated. Evidence was provided by this approach to support the alleviation of negative emotions among nurses and the development of targeted psychological intervention programs. The conceptual framework of this study was illustrated in Figure 1.

## 3 Materials and methods

### 3.1 Study design

This study employed a cross-sectional quantitative design integrating both variable-centered and person-centered approaches to examine the mechanisms underlying the relationships among self-worth, work engagement, emotional labor, and anxiety in nurses. First, latent profile analysis (LPA) was conducted to identify distinct nurse subgroups based on self-worth, work engagement, and the three dimensions of emotional labor. Subsequently, structural equation modeling (SEM) was performed within each subgroup to test the mediation model and evaluate model fit indices and path coefficients. This study followed established empirical paradigms in occupational psychology, and self-administered, anonymous questionnaires were utilized to minimize social desirability bias.

### 3.2 Ethical considerations

This study strictly adhered to the ethical principles outlined in the Declaration of Helsinki and was approved by the Ethics Committee of Wuhan University of Science and Technology (Approval No. 2025142). Prior to the survey, participants were informed of the study's purpose, anonymity, data confidentiality, and their right to withdraw. All participants completed the questionnaires voluntarily and provided informed consent online. The data collected were used solely for scientific research purposes and were not associated with any academic evaluations or administrative decisions. Personal information was anonymized and coded during data processing to ensure privacy protection.

### 3.3 Study setting and participants

From March to August 2025, nurses were recruited from a tertiary grade A general hospital in Wuhan, Hubei Province, using convenience sampling. After obtaining institutional approval, the electronic questionnaire link was distributed via WeChat with the assistance of faculty members and academic advisors. This hospital serves as a major regional healthcare provider with a large nursing workforce, thereby providing a representative setting for examining occupational mental health issues among nurses in high-demand clinical environments. Inclusion criteria were as follows: (1) Clinical nursing staff currently on duty; (2) Voluntary participation in the study and provision of signed informed consent. Exclusion criteria were as follows: (1) Personnel undergoing off-site training or student nurses; (2) Nurses not working in the hospital during the survey period, including those on sick leave, maternity leave, or personal leave. Convenience sampling was justified by practical considerations including accessibility, institutional cooperation, and the need to obtain a sufficiently large sample for latent profile analysis (which requires 300–500 participants for stable profile solutions). The sampling method was deemed appropriate for this exploratory study examining heterogeneous subgroups, as the primary aim was to identify meaningful patterns rather than to estimate population parameters with precise generalizability.

### 3.4 Sample size

To ensure the adequacy of the sample size, *a priori* power analysis was conducted using G^*^Power 3.1 software (57). With a medium effect size (*f* ^2^ = 0.15), significance level α = 0.05, and statistical power 1–β = 0.80, the minimum required sample size was calculated to be 77 participants.

Given that this study also involved LPA and partial least squares structural equation modeling (PLS-SEM), further evaluation of sample size adequacy was necessary. Simulation studies and application guidelines for LPA indicate that when indicators are continuous and between-profile differentiation is high, sample sizes in the range of 300 to 500 are generally sufficient to reliably identify a small number of profiles with acceptable classification accuracy (58). Additionally, the rule of thumb for PLS-SEM requires that the sample size be at least 10 times the maximum number of predictor paths pointing to a single construct (59). In this study, there were four paths directed toward “anxiety,” indicating a minimum requirement of 40 participants. Considering these factors, a target sample size of at least 300 participants was established. The final sample comprised 1,280 valid respondents, fully satisfying all methodological requirements.

### 3.5 Measures

#### 3.5.1 General demographic data questionnaire

A self-developed questionnaire was used to collect demographic and occupational data, including gender, age, department, professional title, educational level, working years, and average monthly income.

#### 3.5.2 Adult self-worth scale

This scale, developed by Luo Yanping in 2010 (60), is a universal instrument for evaluating self-worth in adults. In this study, it was used to assess nurses' self-worth. The scale comprises 28 items across six dimensions: life attitude, personal qualities, physical appearance, interpersonal relationships, social relationships, and family relationships. An example item is “I am a compassionate person.” Items are rated on a 5-point Likert scale ranging from 1 (“Strongly Disagree”) to 5 (“Strongly Agree”). The total score ranges from 28 to 140, with higher scores indicating higher levels of self-worth. The Cronbach's α was 0.899 in this study, indicating good internal consistency.

#### 3.5.3 Generalized anxiety disorder scale (GAD-7)

The GAD-7 scale (61) comprises seven items assessing participants' feelings over the past 2 weeks. Each item is scored from 0 (“Not at all”) to 3 (“Nearly every day”). The total score ranges from 0 to 21, with higher scores indicating greater severity of anxiety symptoms. Scores are interpreted as follows: 0–4 (Minimal anxiety), 5–9 (Mild anxiety), 10–14 (Moderate anxiety), and 15–21 (Severe anxiety). An example item is “Feeling nervous, anxious or on edge”. In this study, the Cronbach's α *f* or the scale was 0.931. The total scale score was adopted for analysis in this study, because all six dimensions converge on the core construct of “an individual's positive self-evaluation” and exhibit high internal consistency among dimensions (average correlation coefficient >0.4). Thus, the total score can more comprehensively reflect an individual's overall level of self-worth.

#### 3.5.4 Utrecht work engagement scale (UWES)

The UWES, developed by Schaufeli et al. (62), measures work engagement across three dimensions: vigor, dedication, and absorption. It consists of 16 items and is widely used among healthcare professionals, teachers, students, and other occupational groups. Items are rated on a 5-point Likert scale, ranging from 1 (“Strongly Disagree”) to 5 (“Strongly Agree”). Higher total scores indicate greater levels of work engagement. The Cronbach's α for the scale in this study was 0.900. The total scale score was used for analysis in this study, as the three factors together constitute the unidimensional construct of “positive work state” and exhibit extremely high internal consistency among themselves, which can comprehensively reflect an individual's overall level of work engagement.

#### 3.5.5 Emotional labor scale (ELS)

This scale, developed by Grandey (45) and translated into Chinese by Luo Hong et al. (63), assesses emotional labor across three dimensions: surface acting, deep acting, and emotional expression requirements. It comprises 14 items rated on a 6-point Likert scale [1 = “Strongly Disagree” to 6 = “Strongly Agree”). The total score ranges from 16 to 96, with higher scores indicating higher levels of emotional labor. An example item is “Required to display emotions that differ from one's genuine feelings”. The Cronbach's α for the total scale and the three dimensions (Surface Acting, Deep Acting, Emotional Expression Requirements) were 0.811, 0.711, 0.826, and 0.872, respectively. Since the three dimensions exhibit an opposing nature in their mechanisms of action—emotional display requirements represent external pressure, surface acting entails resource depletion, and deep acting involves resource gain—this study adopted dimension-specific scores for analysis, which enabled precise differentiation of profile differences across various emotional regulation strategies.

### 3.6 Data analysis

Statistical analyses were performed using SPSS 27.0 for descriptive statistics and Pearson correlations, Mplus 8.3 for latent profile analysis, and SmartPLS 4.1 for PLS-SEM to test the chain mediation model. Bootstrap resampling (5,000 iterations) was applied, and statistical significance was set at The coefficient of determination (R2) and predictive relevance (Q2 ) for endogenous latent variables were reported. Statistical significance was set at *p* < 0.05.

## 4 Results

### 4.1 Descriptive statistics and correlation analysis of variables

The 1,280 nurses were predominantly female (97.7%), aged 31–40 years (41.2%), from internal medicine/surgery departments (35.1% and 28.5%, respectively), holding the titles of staff nurse/senior nurse (49.9% and 38.0%, respectively), with a bachelor's degree (75.2%), having 7–20 years of working experience (66.5%), and earning a monthly income of 4,000–8,000 RMB (77.6%).The descriptive statistics are presented in Table 1.

**Table 1:** Descriptive Statistics and Group Comparisons of Anxiety Scores (*n* = 1280).

Variables	Category	*n* (%)	Anxiety score (M±SD)	*t* / *F*	*p*
Gender	Male	30 (2.3)	7.35 ± 4.92	*t* = 0.31	0.750
	Female	1250 (97.7)	7.11 ± 4.85		
Age	20–30	385 (30.1)	7.58 ± 5.03	*F* = 2.86	0.250
	31–40	528 (41.2)	7.02 ± 4.79		
	41–50	312 (24.4)	6.85 ± 4.67		
	50 or above	55 (4.3)	6.63 ± 4.52		
Department	Internal medicine	449 (35.1)	6.89 ± 4.72	*F* = 3.25	0.006
	Surgery	365 (28.5)	7.23 ± 4.91		
	Obstetrics and gynecology	101 (7.9)	7.05 ± 4.68		
	Pediatrics	72 (5.6)	7.51 ± 5.03		
	ICU	175 (13.7)	7.86 ± 5.12		
	Operating room	115 (9.0)	6.92 ± 4.79		
	Mental health department	3 (0.2)	6.67 ± 4.51		
Professional title	Nurse	137 (10.7)	7.42 ± 4.98	*F* = 0.87	0.630
	Staff nurse	639 (49.9)	7.21 ± 4.89		
	Senior nurse	487 (38.0)	6.95 ± 4.76		
	Associate chief nurse	17 (1.3)	6.78 ± 4.53		
Education	Technical secondary school	10 (0.8)	7.65 ± 5.21	*F* = 1.02	0.440
	Junior college	303 (23.7)	7.32 ± 4.95		
	Bachelor	962 (75.2)	7.05 ± 4.82		
	Master's degree and above	5 (0.4)	6.80 ± 4.67		
Working years	≤ 1Year	9 (0.7)	7.89 ± 5.32	*F* = 1.29	0.170
	2–3 Years	53 (4.1)	7.56 ± 5.01		
	4–6 Years	208 (16.3)	7.28 ± 4.93		
	7–10 Years	455 (35.6)	7.01 ± 4.78		
	11–20 Years	397 (30.9)	6.92 ± 4.81		
	>20 Years	158 (12.4)	6.85 ± 4.53		
Average monthly income	< 2,000 RMB	33 (2.6)	8.25 ± 5.41	*F* = 4.89	0.040
	2,000–4,000 RMB	82 (6.4)	7.86 ± 5.13		
	4,000–6,000 RMB	534 (41.7)	7.23 ± 4.92		
	6,000–8,000 RMB	459 (35.9)	6.89 ± 4.75		
	>8,000 RMB	172 (13.4)	6.51 ± 4.58		

p-values were calculated using the independent samples t-test for comparisons between two groups and one-way analysis of variance (ANOVA) for comparisons among three or more groups.

Table 2 presents the Pearson correlation analysis results for all variables. The findings revealed that nurses' department and average monthly income were correlated with anxiety. Self-worth, work engagement, and deep acting were negatively correlated with anxiety (*p* < 0.01), whereas emotional labor, emotional expression requirements, and surface acting were positively correlated with anxiety (*p* < 0.01). Age, gender, professional title, education level, and years of work experience showed no significant correlation with anxiety (*p* > 0.05). Department and average monthly income, which were correlated with anxiety (*p* < 0.05), were subsequently entered as covariates in the differential analysis.

**Table 2:** Descriptive statistics and correlation analysis of self-worth, work engagement, emotional labor, and anxiety (*n* = 1,280).

Variables	Scores(*M* ± SD)	1	2	3	4	5	6	7
1. Self-worth	78.62 ± 12.35	1	0.56^**^	−0.17^**^	−0.11^*^	−0.32^**^	0.34^**^	−0.39^**^
2. Work engagement	52.37 ± 8.91	0.56^**^	1	−0.15^**^	−0.09^*^	−0.32	0.45	−0.33^**^
3. Emotional labor	56.41 ± 10.24	−0.17^**^	−0.15^**^	1	0.76^**^	0.89^**^	0.33^**^	0.32^**^
4. Emotional expression requirements	18.25 ±4.32	−0.11^**^	−0.09^**^	0.76	1	0.47^**^	0.12^**^	0.19^**^
5. Surface acting	19.63 ± 5.17	−0.32^**^	−0.32^**^	0.89^**^	0.47^**^	1	0.40	0.39^**^
6. Deep acting	18.53 ± 4.86	0.34^**^	0.45^**^	0.33^**^	0.12^**^	0.04	1	−0.09^**^
7. Anxiety	7.12 ± 4.85	−0.39^**^	−0.33^**^	0.32^**^	0.19^**^	0.39^**^	−0.09^**^	1

M ± SD represent mean ± standard deviation.

Pearson correlation coefficients were used to examine bivariate associations among variables.

^**^p < 0.01,

^*^p < 0.05 (two-tailed).

### 4.2 Latent profile analysis results of self-worth, work engagement, and emotional labor

LPA was performed based on the participants' scores on the three dimensions of self-worth, work engagement, and emotional labor, with analyses conducted sequentially starting from the 2-class model; the fit indices of each model are presented in Table 3.

**Table 3:** Fit indices for latent profile analysis models of self-worth, work engagement, and emotional labor.

Model	AIC	BIC	aBIC	Entropy	LM (*p*)	BLRT (*p)*	Latent class proportions
2	17,579.882	17,579.882	17,611.532	0.599	< 0.001	< 0.001	0.546/0.454
**3**	**17,299.305**	**17,412.707**	**17,342.824**	**0.787**	**0.005**	**0.006**	**0.194/0.699/0.107**
4	17,013.674	17,158.003	17,069.062	0.819	0.079	0.082	0.676/0.108/0.181/0.035
5	16,890.872	17,066.129	16,958.128	0.814	0.127	0.130	0.041/0.155/0.662/0.080/0.062

Bold values indicate the optimal fitting model and its corresponding fit indices.

AIC, akaike information criterion; BIC, Bayesian information criterion; aBIC, sample-size adjusted BIC; LMR-LRT, Lo-Mendell-Rubin likelihood ratio test; BLRT, bootstrap likelihood ratio test; Entropy, classification accuracy index.

**Figure 2:**
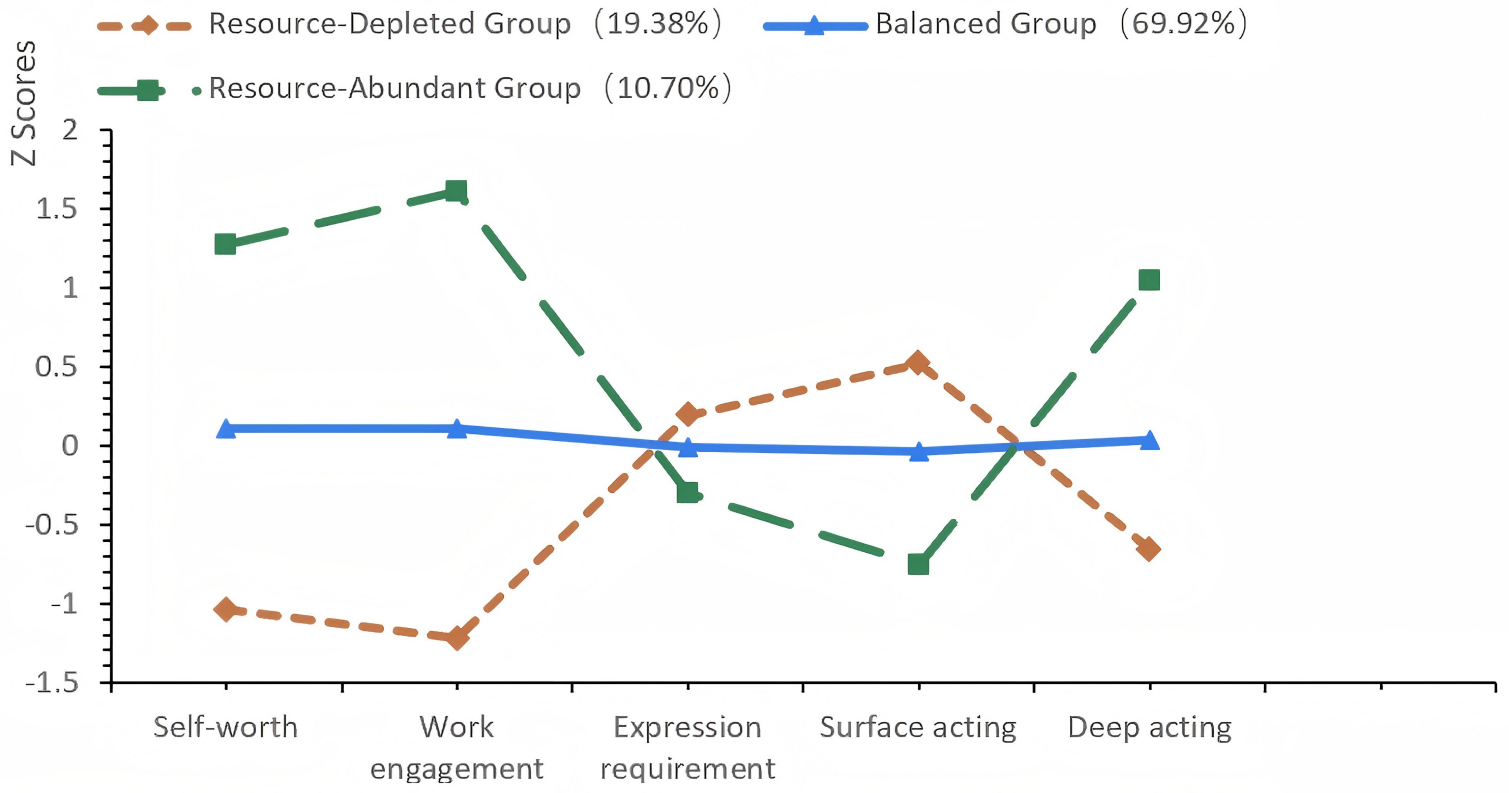
Visualization of joint latent profiles: self-worth, work engagement, and emotional labor.

The results showed that as the number of classes increased, the values of the information evaluation indices AIC, BIC, and aBIC decreased progressively, indicating an improvement in model fit. From the perspective of the LMR and BLRT, the *p*-value for the transition from the 2-class to the 3-class model reached a significant level (*p* < 0.05), demonstrating that the 3-class model was significantly superior to the 2-class model. In contrast, the *p*-value for the transition from the 3-class to the 4-class model was non-significant (*p* > 0.05), suggesting that adding a fourth class had no statistical significance. Additionally, the Entropy value of the 3-class model was close to 0.8, which indicated that the classification accuracy exceeded 90% and the model had high precision. Therefore, the 3-class model was ultimately determined to be the optimal model (Figure 2).

Among the classes, Class 1 was characterized by low self-worth, low work engagement, high emotional display requirements, high surface acting, and low deep acting, and was named the Resource-Depleted Group (19.38%). Class 2 exhibited mean-level scores on self-worth, work engagement, emotional display requirements, surface acting, and deep acting, and was designated the Balanced Group (69.92%). Class 3 was defined by high self-worth, high work engagement, low emotional display requirements, low surface acting, and high deep acting, and was labeled the Resource-Abundant Group (10.70%). This 3-classification result was consistent with the resource distribution pattern of the nursing population: most nurses were in a moderate resource state, while a minority were in extreme resource states.

### 4.3 Measurement model evaluation results

In this study, the coefficient of determination (*R*^2^), predictive relevance (*Q*^2^), and goodness of fit were employed to evaluate the structural model. The explanatory power of the three subgroups for anxiety, as indicated by R^2^, ranged from 0.46 to 0.52, while predictive relevance (*Q*^2^) ranged from 0.31 to 0.38, all exceeding zero, demonstrating good predictive capability of the model. The PLSpredict results showed that the prediction errors (RMSE) of the PLS-SEM model were consistently lower than those of the linear regression benchmark model, further confirming the model's predictive validity. The predictive power indicators for each subgroup's structural model are presented in Table 4. Evaluation of the measurement model revealed that factor loadings for all items ranged from 0.72 to 0.91, all exceeding the recommended threshold of 0.70. Composite reliability (CR) for each latent variable ranged from 0.81 to 0.93, surpassing the 0.70 criterion, and average variance extracted (AVE) ranged from 0.58 to 0.76, all exceeding 0.50, indicating good reliability and convergent validity of the measurement model. Regarding discriminant validity, all heterotrait-monotrait (HTMT) ratios among latent variables were below the critical threshold of 0.85, indicating good discriminant validity among the constructs.

**Table 4:** Predictive power indicators of the structural model across subgroup.

Group	*R*^2^ (anxiety)	*Q*^2^ (anxiety)	PLSpredict result
Resource-depleted group	0.46	0.31	RMSE < LM benchmark
Balanced group	0.52	0.38	RMSE < LM benchmark
Resource-abundant group	0.49	0.33	RMSE < LM benchmark

### 4.4 Chain-mediating effects of work engagement and emotional labor between self-worth and anxiety

Correlation analysis revealed that self-worth, work engagement, and emotional labor were associated with anxiety; however, the variable action patterns may differ among nurses with distinct resource states identified via LPA. After controlling for department and average monthly income, the chain mediating effect of the three dimensions of work engagement and emotional labor between self-worth and anxiety was analyzed.

**Figure 3:**
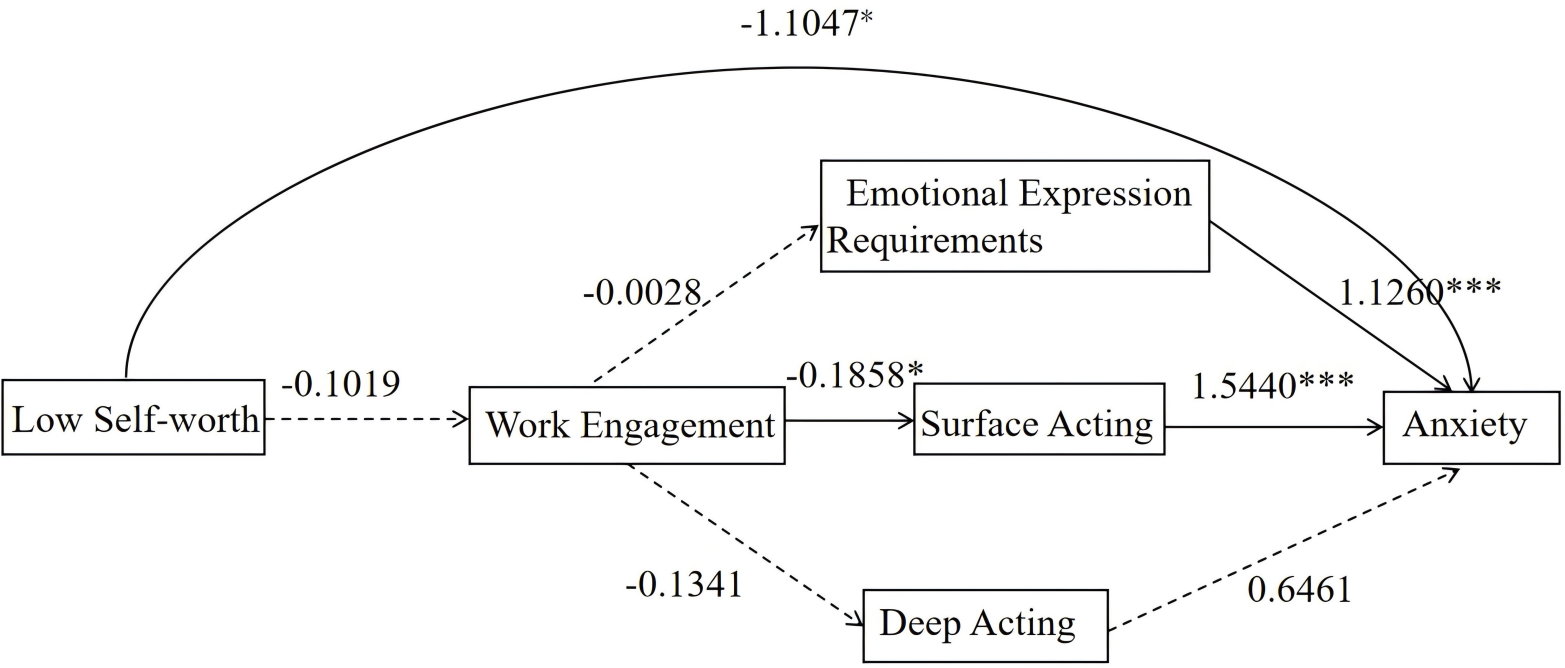
Mediation pathways diagram for the resource-depleted group (19.38%). *p < 0.05; ***p < 0.001. Department and average monthly income were included as covariates.

The results of the mediating effect test indicated that in the Resource-Depletion Group, self-worth exerted a significant direct negative effect on anxiety (β = −1.1047, *p* < 0.05), whereas the chain mediating effect of work engagement and emotional labor was non-significant (the 95% confidence interval (CI) of the total indirect effect included 0), meaning the chain pathway was invalid (Table 5, Figure 3).

**Table 5:** Mediating effects of work engagement and emotional labor in the resource-depleted group (19.38%).

Category	Effect	Path	β	SE	*p*	95%CI	Percentage(%)
	Toal	Low self-worth → anxiety	−1.1047	0.4516	0.015	[−1.9942, −0.2151]	—
Emotional expression requirements dimension	Direct	Low self-worth → anxiety	−1.4124	0.4367	0.001	[−2.2725, −0.5521]	78.21%
	Indirect	Total indirect effect	0.3077	0.2044		[−0.0663, 0.7498]	21.79%
		Low self-worth → work engagement	−0.1019	0.0634	0.114	[−0.2285, 0.0248]	
		Work engagement → emotional expression requirements	−0.0028	0.0900	0.975	[−0.1800, 0.1744]	
		Emotional expression requirements → anxiety	1.1260	0.3044	< 0.001	[0.5265, 1.7256]	
		Work engagement → anxiety	−1.4104	0.4286	0.001	[−2.2547, −0.5661]	
		Low self-worth → emotional expression requirements	0.1454	0.0912	0.112	[−0.0342, −0.0342]	
		Low self-worth → emotional expression requirements	−1.4123	0.4367	0.001	[−2.2725, −0.5521]	
Surface acting dimension	Direct	Low self-worth → anxiety	−1.3739	0.4295	0.002	[−2.2198, −0.5279]	80.41%
	Indirect	Total indirect effect	0.2692	0.2063		[−0.1185, 0.6999]	19.59%
		Low self-worth → work engagement	−0.1019	0.0634	0.114	[−0.2285, 0.0248]	
		Work engagement → surface acting	−0.1858	0.0797	0.021	[−0.3428, −0.0287]	
		Surface acting → anxiety	1.5440	0.3389	< 0.001	[0.8764, 2.2115]	
		Work engagement → anxiety	−1.1267	0.4275	0.009	[−1.9689, −0.2846]	
		Low self-worth → surface acting	0.0811	0.0808	0.317	[−0.0780, 0.2403]	
		Low self-worth → anxiety	−1.3739	0.4295	0.002	[−2.2198, −0.5279]	
Deep acting dimension	Direct	Low self-worth → anxiety	−1.3351	0.4455	0.003	[−2.2125, −0.4576]	82.74%
	Indirect	Total indirect effect	0.2304	0.1583		[−0.0507, 0.5807]	17.26%
		Low self-worth → work engagement	−0.1019	0.0643	0.114	[−0.2285, 0.0248]	
		Work engagement → deep acting	−0.1341	0.0827	0.106	[−2.2969, 0.0287]	
		Deep acting → anxiety	0.6461	0.3379	0.057	[−0.0195, 1.3117]	
		Work engagement → anxiety	−1.3269	0.4396	0.002	[−2.1927, −0.4610]	
		Low self-worth → deep acting	0.1338	0.0838	0.112	[−0.0312, 0.2988]	
		Low self-worth → anxiety	−1.3351	0.4455	0.003	[−2.2125, −0.4576]	

**Figure 4:**
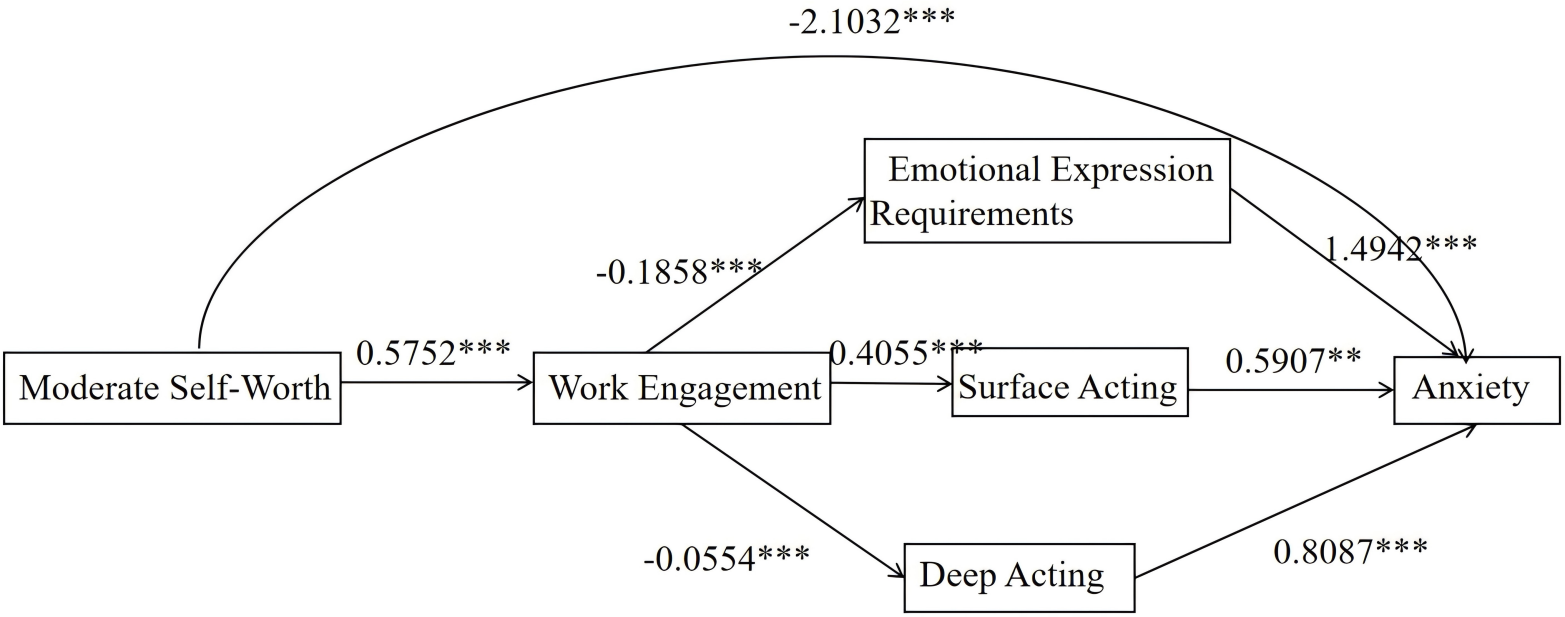
Mediation pathways diagram for the balanced group (69.92%). **p < 0.01; ***p < 0.001. Department and average monthly income were included as covariates.

In the Balanced Group, self-worth exerted a significant negative effect on anxiety (β = −2.1032, *p* < 0.001). Furthermore, self-worth influenced the various dimensions of emotional labor by enhancing work engagement (β = 0.5752, *p* < 0.001) specifically, emotional display requirements (β = −0.1858), surface acting (β = 0.4055), and deep acting (β = −0.0554), all *p* < 0.001 and ultimately exerted an impact on anxiety levels (Table 6, Figure 4).

**Table 6:** Mediating effects of work engagement and emotional labor in the balanced group (69.92%).

Category	Effect	Path	β	SE	*P*	95% CI	Percentage(%)
	Toal	Moderate self-worth → anxiety	−2.1032	0.1641	< 0.001	[−2.4252, −1.7810]	—
Emotional expression requirements dimension	Direct	Moderate self-worth → anxiety	−1.2839	0.1914	< 0.001	[−1.6597, −0.9082]	61.05%
	Indirect	Total indirect effect	−0.8193	0.1397		[−1.0982, −0.5522]	38.96%
		Moderate self-worth → work engagement	0.5752	0.0277	< 0.001	[0.5209, 0.6269]	
		Work engagement → emotional expression requirements	−0.1858	0.0384	< 0.001	[−0.2612, −0.1104]	
		Emotional expression requirements → anxiety	1.4942	0.1634	< 0.001	[1.1735, 1.8149]	
		Work engagement → anxiety	−0.6575	0.1899	0.006	[−1.0302, −0.2847]	
		Moderate self-worth → emotional expression requirements	−0.1883	0.0387	< 0.001	[−0.2643, −0.1123]	
		Moderate self-worth → emotional expression requirements	−1.2839	0.1914	< 0.001	[−1.6597, −0.9082]	
Surface acting dimension	Direct	Moderate self-worth → anxiety	−1.6331	0.1976	< 0.001	[−2.0209, −1.2454]	77.65%
	Indirect	Total indirect effect	−0.4701	0.1358		[−0.7419, −0.2040]	22.35%
		Moderate self-worth → work engagement	0.5752	0.0277	< 0.001	[0.5209, 0.6269]	
		Work engagement → surface acting	0.4055	0.0360	< 0.001	[0.3348, 0.4762]	
		Surface acting → anxiety	0.5907	0.1812	0.001	[0.2350, 0.9464]	
		Work engagement → anxiety	−1.1746	0.2083	< 0.001	[−1.5835, −0.7658]	
		Moderate self-worth → surface acting	0.1149	0.0360	< 0.001	[0.0436, 0.1861]	
		Moderate self-worth → anxiety	−1.6331	0.1976	< 0.001	[−2.0209, −1.2454]	
Deep acting dimension	Direct	Moderate self-worth → anxiety	−1.5229	0.1949	< 0.001	[−1.9054, −1.1403]	72.41%
	Indirect	Total indirect effect	−0.5803	0.1304		[−0.8475, −0.3334]	27.59%
		Moderate self-worth → work engagement	0.5752	0.0277	< 0.001	[0.5209, 0.6296]	
		Work engagement → deep acting	−0.0554	0.0414	< 0.001	[−0.1365, 0.0258]	
		Deep acting → anxiety	0.8087	0.1564	< 0.001	[0.5017, 1.1157]	
		Work engagement → anxiety	−0.8904	0.1934	< 0.001	[−1.2700, −0.5108]	
		Moderate self-worth → deep acting	−0.0525	0.0417	0.208	[−0.1343, 0.0293]	
		Moderate self-worth → anxiety	−1.5229	0.1914	< 0.001	[−1.9054, −1.1403]	

**Figure 5:**
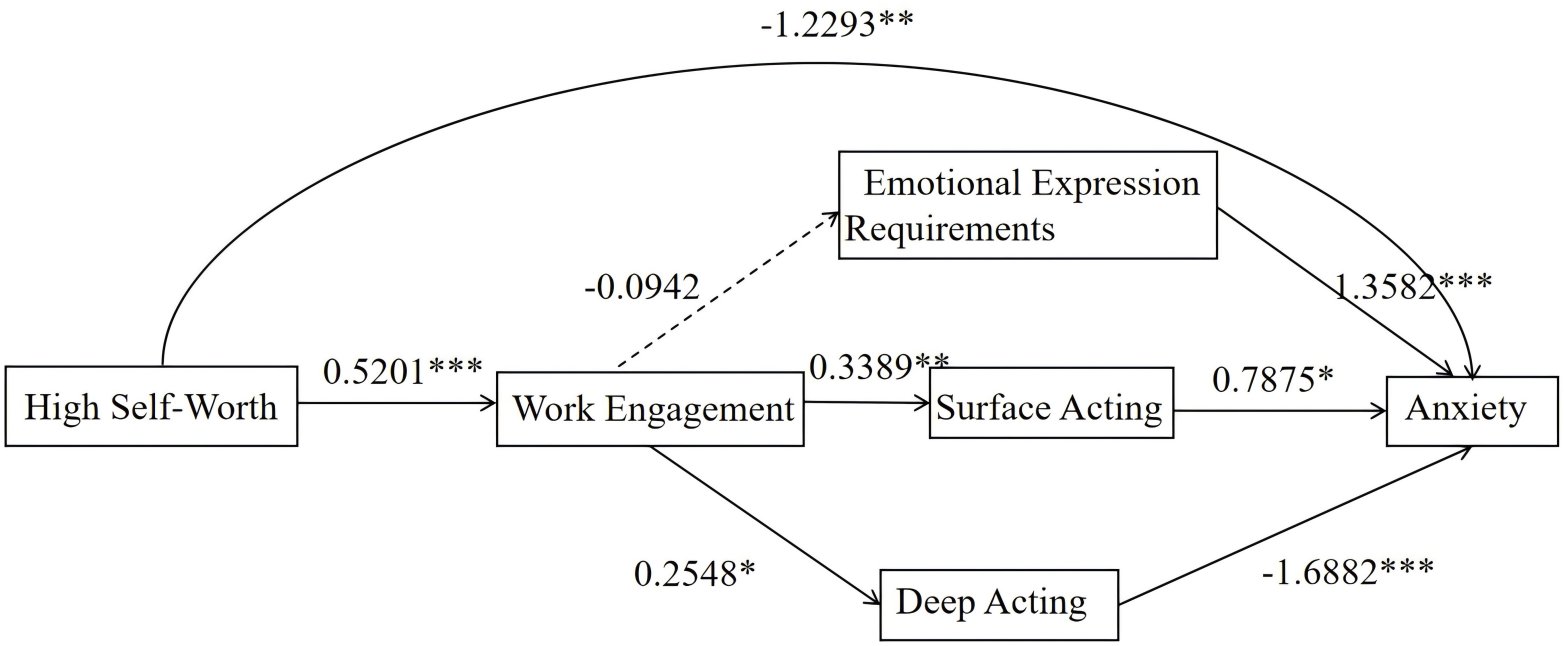
Mediation pathways diagram for the resource-abundant group (10.70%). *p < 0.05; **p < 0.01; ***p < 0.001. Department and average monthly income were included as covariates.

In the Resource-Abundant Group, self-worth significantly and negatively predicted anxiety (β = −1.2293, *p* < 0.01) and influenced anxiety through the chain mediating effects of work engagement and emotional labor. Specifically, self-worth positively predicted work engagement (β = 0.5201, *p* < 0.01), and work engagement positively predicted surface acting (β = 0.3389, *p* < 0.01) and deep acting (β = 0.2548, *p* < 0.05). However, the indirect effect on emotional expression requirements was not significant (β = −0.0942, *p* > 0.05). Additionally, surface acting positively predicted anxiety (β = 0.7875, *p* < 0.05), while deep acting significantly and negatively predicted anxiety (β = −1.6882, *p* < 0.001). These findings indicate that, within the Resource-Abundant Group, self-worth influences nurses' anxiety levels through the chain mediating effects of work engagement and the emotional labor dimensions of surface acting and deep acting (Table 7, Figure 5).

**Table 7:** Mediating effects of work engagement and emotional labor in the resource-abundant group (10.70%).

Category	Effect	Path	β	SE	*p*	95%CI	Percentage (%)
	Toal	High self-worth → anxiety	−1.2293	0.3853	0.002	[−1.9912, −0.4674]	—
Emotional expression requirements dimension	Direct	High self-worth → anxiety	−0.9101	0.4403	0.040	[−1.7810, −0.0393]	74.03%
	Indirect	Total indirect effect	−0.3192	0.2708		[−0.8667, −0.1978]	25.97%
		High self-worth → work engagement	0.5201	0.0711	< 0.001	[0.3795, 0.6607]	
		Work engagement → emotional expression requirements	−0.0942	0.1022	0.083	[−0.2964, 0.1080]	
		Emotional expression requirements → anxiety	1.3582	0.3786	0.001	[0.6093, 2.1070]	
		Work engagement → anxiety	−0.3956	0.4495	0.380	[−1.2847, −0.493]	
		High self-worth → emotional expression requirements	−0.1325	0.0998	0.019	[−0.3299, −0.0645]	
		High self-worth → emotional expression requirements	−0.9101	0.4403	0.041	[−1.7810, −0.0393]	
Surface Acting Dimension	Direct	High self-worth → anxiety	−1.2214	0.4559	0.008	[−2.1232, −0.3197]	99.36%
	Indirect	Total indirect effect	−0.0079	0.2871		[−0.6064, 0.5236]	0.64%
		High self-worth → work engagement	0.5201	0.0711	< 0.001	[0.3795, 0.6607]	
		work engagement → surface acting	0.3389	0.1024	0.001	[0.6607, 0.5415]	
		Surface acting → anxiety	0.7875	0.3899	0.045	[0.0162, 1.5587]	
		Work engagement → anxiety	−0.5346	0.4807	0.268	[−1.4855, 0.4163]	
		High self-worth → surface acting	0.1668	0.1000	0.098	[−0.0310, 0.3645]	
		High self-worth → anxiety	−1.2214	0.4559	0.008	[−2.1232, −0.3197]	
Deep Acting Dimension	Direct	High self-worth → anxiety	0.5201	0.0711	< 0.001	[0.3795, 0.6607]	65.23%
	Indirect	Total indirect effect	−0.4274	0.3032		[−1.0654, −0.1448]	34.77%
		High self-worth → work engagement	0.5201	0.0711	< 0.001	[0.3795, 0.6607]	
		Work engagement → deep acting	0.2548	0.1093	0.021	[0.0386, 0.4710]	
		Deep acting → anxiety	−1.6882	0.3407	< 0.001	[−2.3621, −1.0143]	
		Work engagement → anxiety	−0.1625	0.4398	0.007	[−0.7074, −0.0324]	
		High self-worth → deep acting	0.1707	0.1067	0.011	[0.0404, 0.3818]	
		High self-worth → anxiety	−0.8019	0.4249	0.061	[−1.6423, 0.0385]	

## 5 Discussion

This study aimed to examine the relationship between self-worth and anxiety among nurses, investigate the chain mediating roles of work engagement and emotional labor, and identify heterogeneous subgroups using latent profile analysis.

### 5.1 Heterogeneity in anxiety levels across self-worth/work engagement/emotional labor profiles

Three distinct subgroups of nurses were identified through latent profile analysis: the “Resource-Depleted Group” (19.38%), the “Balanced Group” (69.92%), and the “Resource-Abundant Group” (10.70%). These findings support H5, confirming that self-worth, work engagement, and emotional labor exhibit different combination patterns across individuals, and that mediating effects vary by subgroup. These findings indicate that nurses are not a homogeneous population, with the majority exhibiting moderate levels of anxiety and work-related resources. This moderate distribution may reflect a state of equilibrium within the current nursing work environment and professional development pathways. Specifically, nurses possess sufficient self-worth to sustain basic work motivation and emotional regulation capacity; however, they have not yet reached the stage of high engagement, advanced emotional labor regulation, or fully realized self-worth, suggesting considerable room for optimizing their psychological wellbeing and work performance. The three profiles identified in this study are both consistent with and distinct from previous research on nurse psychological characteristics. Chen (52) reported similar patterns in work engagement profiles among nurses—namely “low-engagement coping,” “moderately balanced,” and “highly engaged focused” types—which align conceptually with our Resource-Depleted, Balanced, and Resource-Abundant groups. However, by incorporating multi-dimensional indicators of self-worth and emotional labor, the present study more precisely captures the core distinctions in “resource states,” reflecting not merely differences in work engagement levels, but synergistic combinations of internal resources (self-worth), behavioral resources (work engagement), and regulatory resources (emotional labor).

Notably, the proportion of the Balanced Group (approximately 70%) exceeded the 58.2% reported by Ma et al. (55) for the moderate-level group in their analysis of work engagement among oncology nurses. This discrepancy may be attributable to differences in study populations: the present sample comprised nurses from a general hospital, where variations in work pressure across departments may predispose the overall resource profile toward a “moderated” distribution. In contrast, specialist nurses, who work in more concentrated and focused clinical environments, tend to exhibit more pronounced subgroup differentiation.

### 5.2 Negative predictive effect of self-worth on anxiety

Self-worth significantly and negatively predicted anxiety across the three subgroups, supporting Hypothesis 1 and previous research (33). However, the strength of this effect varied by subgroup, indicating a “resource-dependent” protective role. In the Resource-Depleted Group, self-worth had a strong direct effect on anxiety, while mediating pathways (work engagement, emotional labor) were invalid, suggesting it served as the sole buffer under severe resource loss. In contrast, in the Balanced and Resource-Abundant Groups, self-worth indirectly reduced anxiety through these mediators, reflecting a synergistic protective effect. These findings align with Sociometer Theory (32), where self-worth's regulatory function intensifies as resources dwindle. Additionally, department type and income were associated with anxiety, implicating that nurses in high-stress units or with lower income may have diminished self-worth, which could exacerbate anxiety and serve as a potential target for tailored interventions.

### 5.3 Variability in the mediating effects of work engagement and emotional labor across different subgroups

According to the study results, the non-significant chain mediation pathway in the “Resource-Depleted Group” revealed the distinctiveness of this subgroup. Consistent with COR Theory, severe resource depletion may lead to “motivation-behavior decoupling,” disrupting the mediating pathways. This finding aligns with previous research (64) on the negative impact of emotional labor on work engagement, suggesting that the possibility of mediation transmission is further weakened under conditions of severe resource depletion. In the “Balanced Group,” work engagement alleviated anxiety by reducing perceived emotional expression requirements, which aligns with the Cognitive Appraisal Theory of Stress (65), indicating that moderately resourced groups can reframe external demands as protective pathways. However, the depleting effect of surface acting maintained anxiety at moderate levels. Deep acting alleviated anxiety by integrating emotional norms into self-identity, while surface acting induced emotional depletion due to professional responsibility. These findings align with Lee (66), who found deep acting reduces anxiety and surface acting increases it among Korean nurses. These results partially support Hypotheses 2, 3, and 4.

In summary, significant differences were observed in the validity, path composition, and direction of effects of the chain mediating effect across the three subgroups, which supports H5.

### 5.4 Strengths and limitations

The study identified latent profiles of nurse resources, expanded the research field of self-worth, and revealed the differential mechanisms through which self-worth, work engagement, emotional labor, and anxiety operate across distinct nurse subgroups, thereby providing nursing managers with evidence for precise intervention. Limitations include a single-center sample and a cross-sectional design that precludes causal inferences.

## 6 Conclusion

Three subgroups with significantly distinct resource states were identified among nurses: the Resource-Depleted Group, the Balanced Group, and the Resource-Abundant Group. Moreover, the effect of self-worth on nurses' anxiety through work engagement and emotional labor was found to be subgroup-dependent.

This finding offers significant guidance for nursing managers in implementing tiered, targeted interventions: for the Resource-Depleted Group, it is essential to closely monitor nurses' work status. When signs of emotional overload are detected, consider temporarily reassigning them from high-demand units (e.g., ICU). Concurrently, implement psychological training programs aimed at enhancing self-worth. For the Balanced Group, develop and promote standardized communication scripts. These tools are designed to reduce surface acting resulting from inappropriate communication; For the Resource-Abundant Group, encourage deep acting through targeted training and guided activities. Establish dedicated spaces for authentic emotional expression within departments, such as “nurse stress relief rooms,” allowing nurses to naturally express emotions during breaks. This facilitates stress release and helps sustain positive emotional labor patterns.

Based on these findings, we recommend strengthening psychological support and improving self-worth among members of the Resource-Depleted Group, optimizing communication strategies to reduce surface acting for the Balanced Group, and encouraging deep emotional regulation for the Resource-Abundant Group. Hospital administrators should develop tailored psychological support programs for each subgroup and regularly assess their effectiveness. Future research may adopt longitudinal designs and multi-center samples to further validate the causal relationships observed in this study.

## Data Availability

The raw data supporting the conclusions of this article will be made available by the authors, without undue reservation.
